# Impact of galectin-3 on neurotrophic factor expression by PCR array: potential implications for the human cornea

**DOI:** 10.3389/fcell.2024.1488877

**Published:** 2024-12-06

**Authors:** Ashley M. Woodward, Pablo Argüeso

**Affiliations:** Department of Ophthalmology, Tufts Medical Center, Tufts University School of Medicine, Boston, MA, United States

**Keywords:** cornea, epithelium, fibroblast, galectin-3, interleukin 6, neurotrophic factors

## Abstract

The cornea is densely innervated to maintain the integrity of the ocular surface, facilitating functions such as sensation and tear production. Following damage, alterations in the corneal microenvironment can profoundly affect its innervation, potentially impairing healing and sensory perception. One protein frequently upregulated at the ocular surface following tissue damage is galectin-3, but its contribution to corneal nerve regeneration remains unclear. Here, we sought to delineate the role of galectin-3 in regulating the expression of neurotrophic factors by different human cell types. Using a pathway-focused PCR array, we first evaluated the expression of neurotrophic factors in primary cultures of human corneal epithelial cells and fibroblasts. We found that these cell types contributed differently to the expression of these factors, with fibroblasts exhibiting higher levels of *nerve growth factor*, *brain-derived neurotrophic factor*, and *GDNF* compared to epithelial cells. Treatment with exogenous galectin-3 did not significantly affect epithelial cells; however, it did lead to increased synthesis and secretion of IL6, a cytokine known to influence neuronal survival and modulate inflammatory responses, by corneal fibroblasts. Using the human-derived SH-SY5Y cell line as a neuron-like cell model, we also found that galectin-3 stimulated the expression of *FOS* and *LIF*, two genes involved in neural differentiation and survival. In summary, these *in vitro* findings suggest that the presence of galectin-3 in the corneal environment may influence the neuronal response to injury.

## Introduction

The cornea is highly innervated, containing a dense network of sensory nerve fibers primarily originating from the ophthalmic branch of the trigeminal ganglion (Muller et al., 2003). These neurons provide mechanical, chemical, and thermal sensitivity to the eye and release numerous trophic factors that maintain the structural and functional integrity of the corneal epithelium (Yang et al., 2018). Loss of corneal innervation can result in neurotrophic keratopathy, a degenerative disease marked by impaired corneal healing and ulceration. Conditions such as dry eye disease, herpetic keratitis, chemical burns, surgical procedures, and diabetes can cause symptoms similar to neurotrophic keratopathy (Versura et al., 2018). In recent decades, novel therapeutic interventions aimed at stimulating corneal nerve regeneration have been proposed for the topical treatment of neurotrophic keratopathy. While these agents promote the anatomical recovery of injured nerves, they present limitations in terms of side effects and their ability to achieve full recovery of nerve sensation. One of the major challenges to achieve full recovery is the complexity of nerve repair, a process that requires precise guidance cues and a supportive microenvironment.

Corneal nerve regeneration is significantly influenced by the synergistic contributions of corneal epithelial cells, fibroblasts, and the extracellular matrix. Corneal epithelial cells produce a variety of neurotrophic factors, such as nerve growth factor (NGF) and brain-derived neurotrophic factor (BDNF), which are essential for the survival, growth, and regeneration of corneal nerves (Sacchetti and Lambiase, 2017). Stromal fibroblasts also contribute to this process by secreting extracellular matrix proteins, cytokines and additional growth factors that stimulate epithelial cell growth and aid in the regeneration of corneal nerves (Yam et al., 2017). These factors collectively create a supportive microenvironment that promotes axonal outgrowth and nerve repair. Dysregulation in the protein expression by these cells can lead to impaired nerve healing and may result in chronic pain or sensory deficits, highlighting the importance of maintaining a balanced expression of neurotrophic and growth factors for effective corneal nerve regeneration.

Galectins are a family of animal lectins that recognize β-galactoside residues on cell surface proteins. Galectin–glycoprotein interactions at the cell surface cause glycoprotein clustering and initiate signaling events that regulate various physiological and pathological processes. Numerous studies have demonstrated an overabundance of galectin-3 in the human and mouse cornea under pathological conditions such as dry eye, ulceration, and herpes simplex virus infection (King et al., 2009; Uchino et al., 2015; Cruzat et al., 2018). Interestingly, the addition of exogenous galectin-3 has been shown to promote epithelial wound healing in the cornea (Cao et al., 2002; Yabuta et al., 2014; Fujii et al., 2015). However, its role in modulating processes associated with corneal innervation remains largely unexplored. In this study, we used a pathway-focused PCR array to examine the effect of exogenous galectin-3 on the expression of neurotrophic factors in different human cell types. Our findings indicate that while galectin-3 has no significant effect on corneal epithelial cells, it does modulate the synthesis and secretion of factors known to influence neural differentiation and survival in corneal fibroblasts and in the SH-SY5Y neuronal cell line.

## Methods

### Preparation of limbal epithelial cell suspensions

Human *postmortem* corneoscleral tissue unsuitable for transplantation was obtained from Lions VisionGift (Portland, OR). The iris root and endothelium were scraped from corneoscleral tissue using a K-sponge spear (Katena). The central portion of the tissue was cut with an 8-mm disposable biopsy punch (Integra Miltex) to produce corneal rims. These corneal rims were subsequently incubated for 1 h at 37°C with 2.4 IU/mL Dispase II (Thermo Fisher Scientific) in a supplemented growth medium (sGMedium) containing a 3:1 mixture of Dulbecco’s Modified Eagle Medium (DMEM) GlutaMAX:F12 with 10% fetal bovine serum (FBS), 0.4 μg/mL hydrocortisone, 5 μg/mL insulin, 1.4 ng/mL triiodothyronine, 24 μg/mL adenine, 8.4 ng/mL cholera toxin, 10 ng/mL epidermal growth factor and 1% antibiotic–antimycotic. The epithelia were scraped with a sterile disposable scalpel no. 10, centrifuged at 400 *g* for 5 min and resuspended in TrypLE Express (Thermo Fisher Scientific) for another 5 min. Cells were then washed with DPBS lacking calcium and magnesium (Thermo Fisher Scientific) and filtered through a 35 µm nylon cell strainer.

### Growth-arrested 3T3-J2 cells

3T3-J2 mouse fibroblasts (Kerafast) were maintained in DMEM GlutaMAX with 10% bovine calf serum and 1% penicillin/streptomycin. Mouse fibroblasts were mitotically inactivated by incubation with 4 μg/mL mitomycin-C (Sigma-Aldrich) for 2 h at 37°C. Cells were then rinsed five times with DPBS lacking calcium and magnesium, detached with TrypLE Express and frozen in DMEM GlutaMAX supplemented with 20% bovine calf serum and 10% dimethylsulfoxide (DMSO) for 2 h at −80°C before cryopreservation in liquid nitrogen.

### *Ex vivo* expansion and culture of human limbal epithelial cells

Growth-arrested 3T3-J2 fibroblasts were plated at a density of 4.6 × 10^4^/cm^2^ in a 100-mm tissue culture dish. On the following day, limbal epithelial cell suspensions were resuspended in sGMedium and seeded at a density of up to 6,000 cells/cm^2^ in 100-mm tissue culture dishes containing growth-arrested 3T3-J2 fibroblasts. The sGMedium was changed on the third day and every other day thereafter. After 7 days, the 3T3-J2 feeder cells were detached using Versene (Thermo Fisher Scientific) for approximately 1 min, followed by a wash in DPBS and a 10-min incubation with TrypLE Express to detach the epithelial cells. These cells were pelleted by centrifugation and frozen (passage 0) in DMEM GlutaMAX supplemented with 20% fetal bovine serum and 10% DMSO or further passaged on a fresh 3T3-J2 feeder layer. At passage 1, limbal epithelial cells were plated at a density of 3 × 10^3^/cm^2^. Once cultures reached 90% confluence, cells were treated with either 3 µM recombinant human galectin-3 (PeproTech) or bovine serum albumin.

### *Ex vivo* expansion and culture of human corneal fibroblasts

After removal of both the epithelium and endothelium by scraping with a razor blade, primary corneoscleral tissue was cut into small pieces and explants allowed to adhere to the bottom of tissue culture plates in the presence of DMEM/F12 supplemented with 10% newborn calf serum. Fibroblasts were passaged after 1–2 weeks of cultivation. At passage one to 4, fibroblasts were plated at a density of 5 × 10^4^/cm^2^. Once cultures reached 90% confluence, cells were treated with either 3 µM recombinant human galectin-3 or bovine serum albumin.

### Culture of the human neuroblastoma cell line SH-SY5Y

Human neuroblastoma SH-SY5Y cells (ATCC) were cultured, as previously described (Bufalo et al., 2022), in DMEM/F12 supplemented with 10% FBS and 1% penicillin/streptomycin in a humidified atmosphere of 5% CO_2_ at 37°C. Cells were cultured to about 90% confluence. Differentiation was induced first by switching to DMEM/F12 supplemented with 2% FBS, 10 µM all-trans retinoic acid (Thermo Fisher Scientific) and 1% penicillin/streptomycin until day 5 and then switching to serum-free DMEM/F12 supplemented with 50 ng/mL human BDNF (R&D Systems) and 1% penicillin/streptomycin for 48 h. On day 7 of differentiation, neurons were treated with either 3 µM recombinant human galectin-3 or bovine serum albumin.

### RNA isolation and cDNA synthesis

Total RNA was isolated from cultures using the RNeasy Micro Kit (Qiagen) following the manufacturer’s instructions. Residual genomic DNA in the RNA preparation was eliminated by on-column DNase I digestion (Qiagen). Total RNA was transcribed using the RT^2^ First Strand Kit for PCR array and iScript™ cDNA Synthesis Kit for qPCR.

### Human neurotrophins and receptors PCR array

Analysis of genes encoding neurotrophic factors was carried out using a Human Neurotrophin and Receptors PCR Array (RT^2^ Profiler™ PCR array, Qiagen) according to the manufacturer’s instructions. The ΔΔCT and 2^−ΔΔCT^ methods were used for the relative quantitation of the number of transcripts. Fold changes were calculated after normalizing to the mean of *ACTB*, *B2M*, *GAPDH*, *HPRT1*, and *RPLP0*. Data were analyzed using Qiagen’s web-based PCR array data analysis software.

### qPCR

The qPCR was performed using the SsoAdvanced™ Universal SYBR^®^ Green Supermix (Bio-Rad). Primer sequences for *IL6* (Unique Assay ID qHsaCID0020314), *FOS* (Unique Assay ID qHsaCED0046695), *LIF* (Unique Assay ID qHsaCED0037597) and *GAPDH* (Unique Assay ID qHsaCED0038674) mRNA were obtained from Bio-Rad. Gene expression was measured in a Bio-Rad CFX Connect thermocycler with the following parameters: 2 min at 95°C, followed by 40 cycles of 5 s at 95°C and 30 s at 60°C. Fold changes were calculated using the comparative ΔΔCT method by normalizing to *GAPDH*.

### ELISA

Cell culture medium was centrifuged to remove cellular debris and equal volumes of supernatant used to carry out ELISA for IL6 and LIF according to the manufacturer’s protocol (Thermo Fisher Scientific).

### Statistical analysis

Analysis was performed in GraphPad Prism 10. All experiments were performed in biological replicates of three or more. Values are graphed as box-and-whisker plots showing the 25th and 75th percentiles (boxes), the median, and the minimum and maximum data values (whiskers). Significance was determined for primary cultures using the *t*-test and for the SH-SY5Y cell line using the *t*-test or Mann–Whitney test. Significance was established as *p < 0.05 and **p < 0.01.

## Results and discussion

### Neurotrophic factor expression in human corneal cells

**FIGURE 1:**
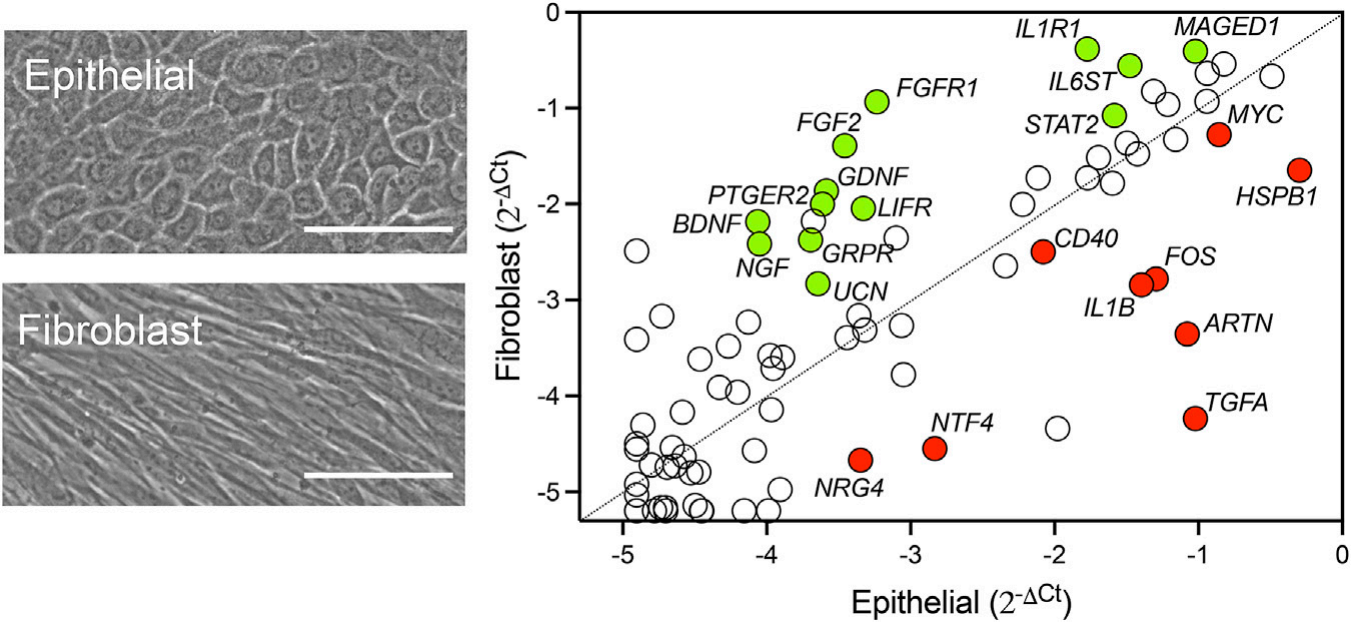
Relative expression of neurotrophic factors in human corneal epithelial cells and fibroblasts. Representative phase-contrast images of primary cultures of human corneal epithelial cells and fibroblasts. Scale bars: 100 µm. The relative transcript abundance of genes encoding secreted neurotrophins and receptors was determined by using the RT^2^ Profiler™ PCR Array Human Neurotrophins and Receptors. The scatterplot shows gene expression normalized to the average C_t_ of housekeeping genes using the comparative 2^−ΔΔCT^ method. Statistically significant upregulated genes in fibroblasts and epithelial cells are shown in green and red, respectively. Statistical significance was determined using the unpaired *t*-test (*p* < 0.05, n = 3 human donors/cell type).

Neurotrophic factors produced by the cornea play a crucial role in promoting nerve regeneration by supporting neuronal survival, growth, and differentiation. These factors help restore corneal innervation, which is essential for maintaining corneal sensitivity and health. Using a pathway-focused PCR array to examine the transcriptional levels of known neurotrophic factors in cultured human corneal epithelial cells and corneal fibroblasts, we found that these cell types contributed differently to the expression of these factors (Figure 1; Supplementary Table S1). By directly comparing relative mRNA levels, we observed that neurotrophin-4 (*NTF4*) was predominantly expressed by epithelial cells, whereas *NGF*, *BDNF*, and glial cell line-derived neurotrophic factor (*GDNF*) were primarily expressed in fibroblasts. Consistent with these results, a previous study detected *NT4* transcription only in cultured human corneal epithelial cells, while *GDNF* transcription was found exclusively in cultured stromal fibroblasts (You et al., 2000). However, contrary to our findings, the authors reported similar expression levels of NGF and BDNF between the cell types. This discrepancy may be attributed to differences in culture media conditions and the use of a transformed epithelial cell line in their experiments. Other genes we found in the array that are known to be exclusively expressed by epithelial cells and influence corneal nerve regeneration included interleukin-1β (*IL1B*) and transforming growth factor α (*TGFA*) (Li and Tseng, 1995).

In addition, we identified several highly expressed factors whose roles in corneal nerve health have not yet been fully explored. One of these factors is artemin (ARTN), a member of the GDNF family of ligands and the most highly expressed neurotrophin in our corneal epithelial cell culture (Figure 1). Artemin can be upregulated during inflammation and supports the survival of various peripheral neuron populations through its glycosylphosphatidylinositol-anchored receptor GFRα3 (Baloh et al., 1998). Interestingly, overexpression of artemin in the skin enhances the expression of the ion channel TRPV1 (Elitt et al., 2006), a protein known for its crucial role in the progression of dry eye by promoting inflammation and the development of hyperalgesia (Gou et al., 2024). Although GFRα3 is present on specific subpopulations of corneal nerves, there is currently no information on how artemin/GFRα3 signaling affects corneal function (Alamri et al., 2015).

### Role of galectin-3 in regulating neurotrophic factor expression in human corneal cells

In the central nervous system, galectin-3 is recognized for its role in promoting pro-inflammatory responses while also playing a crucial part in the recovery phase of neurodegenerative diseases by modulating neuronal activity and glial cell function (Nio-Kobayashi and Itabashi, 2021). However, its role within the peripheral nervous system is less well understood. Galectin-3 appears to have dual functions in peripheral nerve injury. On one hand, it facilitates neuronal cell adhesion and neurite outgrowth in dorsal root ganglion cultures, indicating a supportive role in nerve regeneration (Pesheva et al., 1998). On the other hand, studies on galectin-3 knockout mice with sciatic nerve injury show accelerated regeneration due to enhanced phagocytic activity in Schwann cells and macrophages (Narciso et al., 2009; Mietto et al., 2013). These findings suggest that galectin-3 may have opposing effects on peripheral nerve injury, depending on its interaction with specific cell types.

**FIGURE 2:**
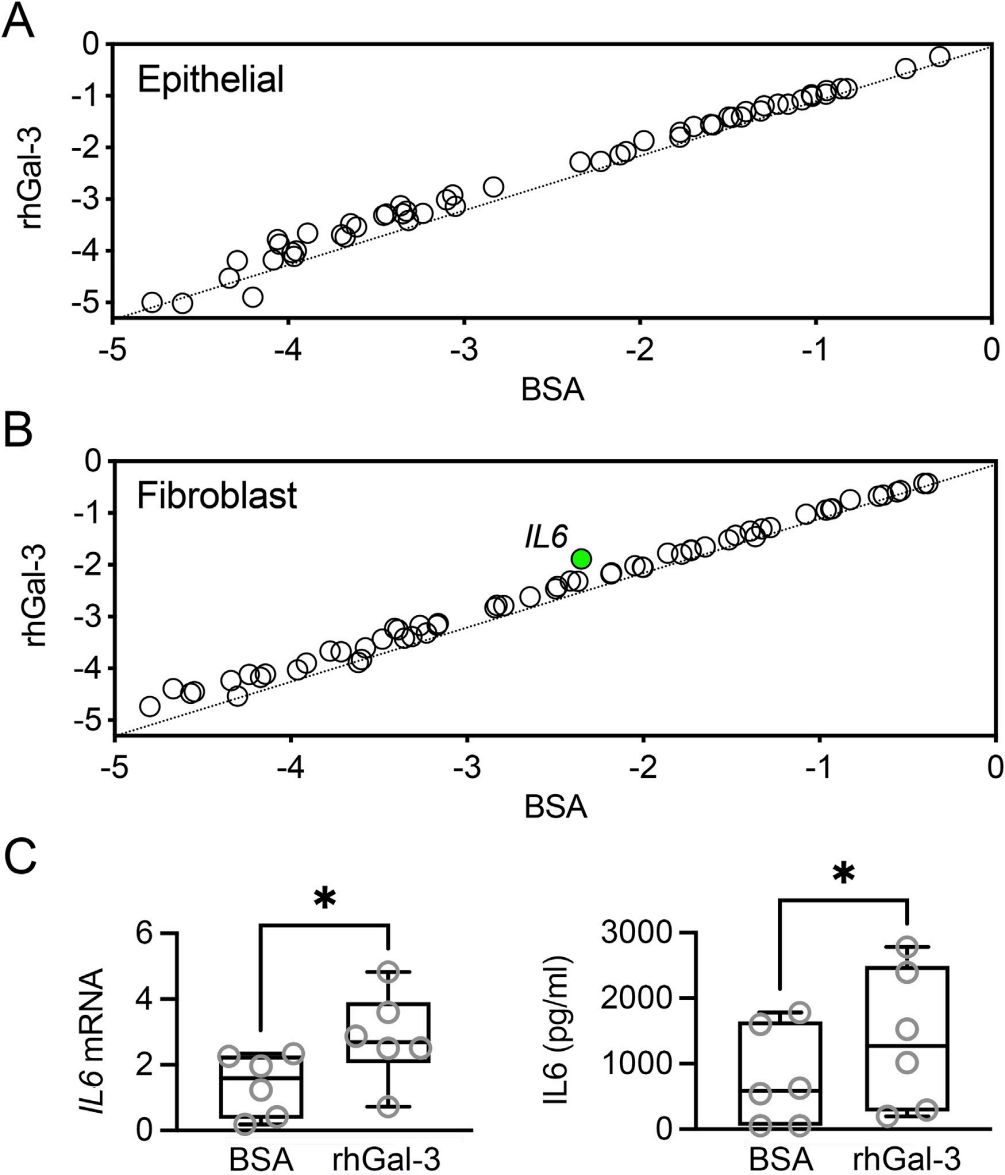
Effect of recombinant human galectin-3 (rhGal-3) on neurotrophic factor expression in human corneal epithelial cells and fibroblasts. Human primary corneal epithelial cells **(A)** and fibroblasts **(B)** were treated with 3 µM rhGal-3 or bovine serum albumin (BSA) for 48 h (n = 3 human donors/cell type). The expression of neurotrophins and receptors was assessed by using a RT^2^ Profiler™ PCR Array. Treatment with rhGal-3 resulted in a statistically significant upregulation of *IL6* in fibroblasts (green). **(C)** The upregulation of *IL6* expression in human corneal fibroblasts was confirmed by qPCR. Levels of IL6 in the conditioned media were quantified by ELISA. Statistical significance was determined using the unpaired *t*-test (B, *p* < 0.05, n = 3 human donors) and the paired *t*-test (C, n = 6 human donors). *p < 0.05.

Galectin-3 levels increase in the injured cornea, where it plays a complex role in the wound healing process. After corneal injury, galectin-3 is upregulated and contributes to both inflammation and tissue repair by modulating the activity of various cell types, including epithelial cells and fibroblasts. In this study, we explored whether galectin-3 influences the expression of factors produced by these cells that are critical for the growth, survival, and maintenance of neurons. Pathway-focused PCR array analysis revealed that incubating human corneal epithelial cells with recombinant human galectin-3 (rhGal-3) for 48 h did not result in significant changes in the expression of neurotrophic factors (Figure 2A). It is important to recognize that PCR arrays may have limitations in detecting low-abundance transcripts or subtle changes in gene expression that could still be biologically significant. Additionally, the limited gene coverage of the array and the possibility that gene expression changes might occur at time points not captured in the study should also be considered.

In human corneal fibroblasts, incubation with rhGal-3 resulted in a statistically significant increase in interleukin-6 (*IL6*) levels compared to the bovine serum albumin control (Figure 2B). The increase in mRNA levels and protein secretion for IL6 was validated using qPCR and ELISA, respectively (Figure 2C). The impact of galectin-3 to IL6 secretion has been previously established in an *in vivo* model of *Aspergillus fumigatus* infection (Niu et al., 2021). In that study, subconjunctival injection with recombinant mouse galectin-3 increased IL6 levels, while injection with galectin-3 siRNA resulted in decreased IL6 production following corneal infection. The authors further investigated the influence of galectin-3 stimulation in polymorphonuclear leukocytes, but did not explore other potential cellular targets. Our data derived from human cells indicate that fibroblasts also play a role in the upregulation of IL6. IL6 can act as a neurotrophic cytokine, promoting neurite regeneration from transected nerve terminals of rat dorsal root ganglia (Shuto et al., 2001). However, the role of IL6 *in vivo* is complex due to its immune-related activities. For instance, in a mouse model of sterile corneal injury, IL6 neutralization preserved corneal nerves, whereas IL6 supplementation aggravated nerve damage and increased myeloid cell recruitment (Lee et al., 2023). The complexity of the galectin-3/IL6 axis highlights the need for further research to clarify how this pathway influences corneal innervation.

### Role of galectin-3 in regulating neurotrophic factor expression in SH-SY5Y cells

**FIGURE 3:**
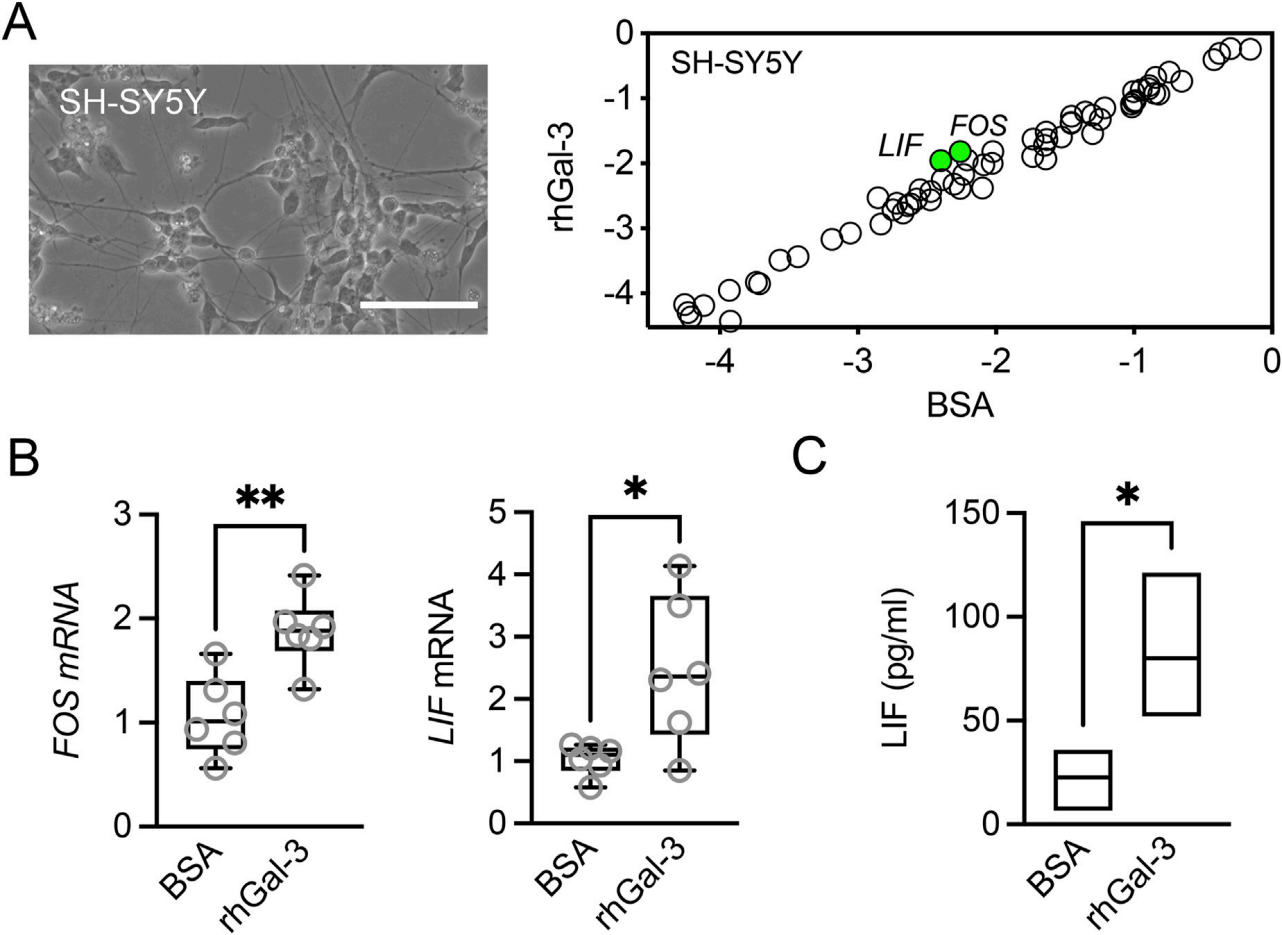
Effect of recombinant human galectin-3 (rhGal-3) on neurotrophic factor expression in human SH-SY5Y cells**. (A)** Representative phase-contrast image of differentiated human SH-SY5Y cells. Scale bar: 100 µm. Cells were treated with 3 µM rhGal-3 or bovine serum albumin (BSA) for 48 h. The expression of neurotrophins and receptors was assessed by using the RT^2^ Profiler™ PCR Array. Treatment with rhGal-3 resulted in a statistically significant upregulation of *FOS* and *LIF* (green). **(B)** The upregulation of *FOS* and *LIF* expression was confirmed by qPCR. **(C)** Levels of LIF in the conditioned media were quantified by ELISA. Statistical significance was determined using the unpaired *t*-test in A (*p* < 0.05, n = 3 independent experiments) and C (n = 3 independent experiments), and the Mann-Whitney test for nonparametric data (B, n = 6 independent experiments). *p < 0.05; **p < 0.01.

To explore the potential direct effects of galectin-3 on neurons, we employed the human-derived SH-SY5Y neuroblastoma cell line. Differentiation of these cells with retinoic acid and BDNF has been shown to promote the expression of neuronal markers, along with sensory neuron markers essential for pain sensation (Bufalo et al., 2022). Transcriptional analysis using the pathway-focused PCR array revealed that SH-SY5Y cells predominantly express a distinct subset of genes, including *VGF*, *NTRK2*, *CNTFR*, *BCL2*, and *NPY*, compared to epithelial cells and fibroblasts (Supplementary Table S1). Treatment of SH-SY5Y cells with rhGal-3 led to the upregulation of *FOS*, a marker of neuronal activity that is rapidly induced in response to various cellular stimuli, and *LIF*, a cytokine belonging to the IL6 family (Figure 3A). The transcriptional upregulation of these factors was confirmed by qPCR, while the secretion of LIF was verified using ELISA (Figures 3B, C). LIF has been shown to promote elongating but not arborizing neurite outgrowth *in vitro*, and it is essential for the normal regeneration of injured adult sensory neurons *in vivo* (Cafferty et al., 2001). In the rabbit cornea, the application of LIF eye drops following LASIK surgery led to enhanced nerve regeneration and reduced the incidence of dry eye symptoms (Pan et al., 2011). These data suggest that overabundance of galectin-3 in the human cornea under pathological conditions may enhance the expression of regenerative genes in corneal nerves.

## Conclusion

The findings of this *in vitro* study highlight the distinct contributions of corneal epithelial cells and fibroblasts to the expression of neurotrophic factors within the cornea. Specifically, epithelial cells and fibroblasts appear to differentially regulate the production of these factors, which are crucial for supporting nerve health and regeneration. Additionally, the study suggests that galectin-3, a protein present in the corneal environment, may play a role in modulating the neuronal response to injury.

## Data Availability

The datasets presented in this study can be found in Supplementary Table S1 and the NCBI Gene Expression Omnibus database (accession number GSE276090).
