# Supplementary material for: Impact of galectin-3 on neurotrophic factor expression by PCR array: potential implications for the human cornea
Source: Front Cell Dev Biol. 2024 Dec 6;12:1488877. doi: 10.3389/fcell.2024.1488877 (PMC11660451; doi:10.3389/fcell.2024.1488877)
Supplement: Supplementary file 1 [file Table1.docx]

| **Supplemental Table S1.** Relative expression (ΔC_T_) of genes encoding neurotrophic factors in human corneal cell types. High positive ΔC_T_ values reflect low amplification efficiency. The values in bold indicate that gene expression was significantly different in fibroblast cultures compared to epithelial cultures (2^-ΔCT^, p<0.05). | | | | | |
| --- | --- | --- | --- | --- | --- |
| **Gene Symbol** | **RefSeq*** | **Relative Expression (ΔC_T_)**  **Epithelial** | **Relative Expression (ΔC_T_)**  **Fibroblast** | **Relative Expression (ΔC_T_)**  **SH-SY5Y** | **Fibroblast/Epithelial Fold Change (2^-ΔCT^)** |
| *ADCYAP1R1* | NM_001118 | 11.42 | 12.28 | 5.50 | 0.553 |
| ***ARTN*** | NM_001136215 | 3.60 | 11.25 | 7.51 | **0.005** |
| *BAX* | NM_004324 | 4.04 | 3.23 | 2.80 | 1.758 |
| *BCL2* | NM_000633 | 11.42 | 11.97 | 2.97 | 0.682 |
| ***BDNF*** | NM_001709 | 11.42 | 7.34 | 9.50 | **16.904** |
| *BEX3* | NM_014380 | 2.74 | 1.82 | 0.53 | 1.890 |
| *CBLN1* | NM_004352 | 11.42 | 12.28 | 6.75 | 0.553 |
| *CCKAR* | NM_000730 | 11.42 | 12.28 | 9.05 | 0.553 |
| ***CD40*** | NM_001250 | 6.93 | 8.32 | 11.74 | **0.379** |
| *CNTF* | NM_000614 | 11.03 | 11.28 | 8.22 | 0.840 |
| *CNTFR* | NM_001842 | 11.42 | 12.28 | 2.96 | 0.553 |
| *CRH* | NM_000756 | 11.42 | 12.28 | 11.74 | 0.553 |
| *CRHBP* | NM_001882 | 11.42 | 12.28 | 11.74 | 0.553 |
| *CRHR1* | NM_004382 | 11.42 | 12.28 | 8.56 | 0.553 |
| *CRHR2* | NM_001883 | 11.42 | 12.28 | 11.74 | 0.553 |
| *CX3CR1* | NM_001337 | 11.42 | 12.28 | 11.74 | 0.553 |
| *CXCR4* | NM_003467 | 11.42 | 12.28 | 5.75 | 0.553 |
| *FAS* | NM_000043 | 5.88 | 5.79 | 7.34 | 1.068 |
| ***FGF2*** | NM_002006 | 11.28 | 4.78 | 5.44 | **90.553** |
| *FGF9* | NM_002010 | 11.42 | 12.28 | 11.74 | 0.553 |
| ***FGFR1*** | NM_015850 | 10.73 | 3.14 | 4.03 | **192.930** |
| ***FOS*** | NM_005252 | 4.33 | 9.60 | 7.51 | **0.026** |
| *FRS2* | NM_006654 | 7.40 | 6.68 | 4.83 | 1.652 |
| *FUS* | NM_004960 | 3.87 | 4.42 | 1.00 | 0.684 |
| *GALR1* | NM_001480 | 11.42 | 12.28 | 11.74 | 0.553 |
| *GALR2* | NM_003857 | 11.42 | 12.28 | 11.74 | 0.553 |
| ***GDNF*** | NM_000514 | 11.42 | 6.23 | 11.74 | **36.529** |
| *GFRA1* | NM_005264 | 11.42 | 8.68 | 4.34 | 6.693 |
| *GFRA2* | NM_001495 | 11.42 | 12.28 | 7.42 | 0.553 |
| *GFRA3* | NM_001496 | 11.42 | 12.28 | 5.77 | 0.553 |
| *GMFB* | NM_004124 | 4.97 | 4.55 | 3.34 | 1.342 |
| ***GRPR*** | NM_005314 | 11.42 | 7.93 | 10.77 | **11.214** |
| *HCRT* | NM_001524 | 11.42 | 12.28 | 11.74 | 0.553 |
| ***HSPB1*** | NM_001540 | 1.03 | 5.51 | 4.88 | **0.045** |
| *IL10* | NM_000572 | 11.42 | 12.28 | 11.74 | 0.553 |
| *IL10RA* | NM_001558 | 11.42 | 12.28 | 11.29 | 0.553 |
| ***IL1B*** | NM_000576 | 4.65 | 10.71 | 11.74 | **0.015** |
| ***IL1R1*** | NM_000877 | 5.98 | 1.52 | 11.74 | **22.123** |
| *IL6* | NM_000600 | 10.52 | 8.18 | 11.74 | 5.062 |
| *IL6R* | NM_000565 | 7.88 | 8.80 | 11.62 | 0.528 |
| ***IL6ST*** | NM_002184 | 4.91 | 1.87 | 3.15 | **8.218** |
| *LIF* | NM_002309 | 7.05 | 7.09 | 7.97 | 0.968 |
| ***LIFR*** | NM_002310 | 10.75 | 6.85 | 4.49 | **14.914** |
| ***MAGED1*** | NM_006986 | 3.44 | 1.36 | 1.39 | **4.235** |
| *MC2R* | NM_000529 | 11.42 | 12.28 | 11.74 | 0.553 |
| *MEF2C* | NM_002397 | 11.42 | 10.72 | 7.94 | 1.622 |
| *MT3* | NM_005954 | 11.42 | 12.28 | 11.74 | 0.553 |
| ***MYC*** | NM_002467 | 2.86 | 4.29 | 3.31 | **0.371** |
| *NF1* | NM_000267 | 5.64 | 5.04 | 3.38 | 1.521 |
| ***NGF*** | NM_002506 | 11.42 | 8.10 | 6.72 | **9.962** |
| *NGFR* | NM_002507 | 6.89 | 12.28 | 7.66 | 0.024 |
| *NPFF* | NM_003717 | 11.42 | 11.96 | 9.42 | 0.688 |
| *NPFFR2* | NM_053036 | 11.42 | 12.28 | 5.07 | 0.552 |
| *NPY* | NM_000905 | 11.42 | 12.28 | 4.32 | 0.553 |
| *NPY1R* | NM_000909 | 11.42 | 12.28 | 11.74 | 0.553 |
| *NPY2R* | NM_000910 | 11.42 | 12.28 | 8.89 | 0.553 |
| *NPY4R* | NM_005972 | 11.42 | 11.99 | 10.17 | 0.674 |
| *NR1I2* | NM_022002 | 11.42 | 12.28 | 11.74 | 0.553 |
| *NRG1* | NM_013957 | 5.31 | 7.42 | 5.43 | 0.232 |
| *NRG2* | NM_013982 | 11.42 | 12.28 | 9.13 | 0.553 |
| ***NRG4*** | NM_138573 | 10.98 | 12.28 | 11.74 | **0.407** |
| *NTF3* | NM_002527 | 11.42 | 11.34 | 11.74 | 1.058 |
| ***NTF4*** | NM_006179 | 9.41 | 12.28 | 11.74 | **0.138** |
| *NTRK1* | NM_002529 | 11.42 | 12.28 | 8.78 | 0.553 |
| *NTRK2* | NM_006180 | 11.42 | 11.06 | 2.82 | 1.282 |
| *NTSR1* | NM_002531 | 10.25 | 12.08 | 11.74 | 0.282 |
| *PNOC* | NM_006228 | 11.42 | 12.28 | 11.74 | 0.553 |
| *PSPN* | NM_004158 | 10.93 | 11.11 | 8.22 | 0.887 |
| ***PTGER2*** | NM_000956 | 11.42 | 6.84 | 8.52 | **23.934** |
| *STAT1* | NM_007315 | 4.37 | 2.95 | 3.33 | 2.669 |
| ***STAT2*** | NM_005419 | 5.28 | 3.61 | 4.14 | **3.194** |
| *STAT3* | NM_003150 | 3.13 | 2.19 | 2.48 | 1.918 |
| *STAT4* | NM_003151 | 10.11 | 10.94 | 6.97 | 0.563 |
| *TACR1* | NM_001058 | 11.42 | 11.84 | 11.74 | 0.751 |
| *TFG* | NM_006070 | 3.13 | 3.10 | 2.72 | 1.020 |
| ***TGFA*** | NM_003236 | 3.41 | 12.28 | 11.74 | **0.002** |
| *TGFB1* | NM_000660 | 1.64 | 2.22 | 4.84 | 0.671 |
| *TP53* | NM_000546 | 4.73 | 4.91 | 2.18 | 0.887 |
| *TRO* | NM_016157 | 11.42 | 7.48 | 3.38 | 15.351 |
| ***UCN*** | NM_003353 | 11.42 | 9.47 | 5.45 | **3.855** |
| *VGF* | NM_003378 | 11.42 | 11.43 | 1.27 | 0.989 |
| *Reference sequence database at NCBI (https://www.ncbi.nlm.nih.gov/refseq/) | | | | | |
